# Bis(chloro­acetato)-κ^2^ *O*,*O*′;κ*O*-methanol-κO-bis­(2-methyl­furo[3,2-*c*]pyridine-κ*N*)copper(II)

**DOI:** 10.1107/S1600536808008404

**Published:** 2008-04-02

**Authors:** Dušan Mikloš, Jozef Miklovič, Viera Mrázová, Jan Moncol, Peter Segľa

**Affiliations:** a Department of Inorganic Chemistry, Slovak Technical University, Radlinského 9, SK-812 37, Bratislava, Slovakia; b Department of Chemistry, Faculty of Natural Science, University of St. Cyril and Methodius, SK-91701 Trnava, Slovakia

## Abstract

In the title compound, [Cu(C_2_H_2_ClO_2_)_2_(C_8_H_7_NO)_2_(CH_4_O)], the Cu^2+^ ion has a highly distorted square-bipyramidal (4 + 1 + 1) coordination environment and is bonded to three carboxyl­ate O atoms of two chloro­acetate anions (monodentate and asymmetrically bidentate), two pyridine N atoms of 2-methyl­furo[3,2-*c*]pyridine and one methanol O atom. There is an intra­molecular O—H⋯O hydrogen bond. Inter­molecular C—H⋯O hydrogen bonds result in the formation of a three-dimensional network and π–π stacking inter­actions [3.44–3.83 Å] are observed between symmetry-related rings of 2-methyl­furo[3,2-*c*]pyridine. Further inter­actions in the crystal structure are a short Cl⋯Cl inter­action [3.384 (2)Å] and C—H⋯π inter­actions between 2-methyl­furo[3,2-*c*]pyridine rings.

## Related literature

For general background, see: Desiraju (1995 ▶); Janiak (2000 ▶); Suezawa *et al.* (2002 ▶). For related literature, see: Baran *et al.* (2005 ▶); Eloy & Deryckere (1971 ▶); Ivaniková *et al.* (2006 ▶); Mikloš *et al.* (2005 ▶); Miklovič *et al.* (2004 ▶); New *et al.* (1989 ▶); Segľa *et al.* (2005 ▶); Titiš *et al.* (2007 ▶); Vrábel *et al.* (2007*a* ▶,*b* ▶). For similar structures, see: Borel *et al.* (1978 ▶); Moncol *et al.* (2007 ▶); Wang *et al.* (2005 ▶).
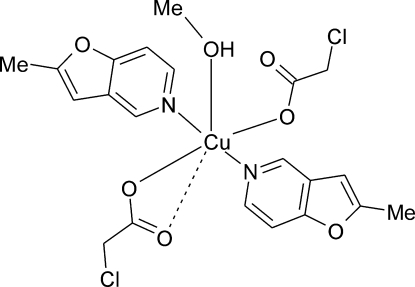


## Experimental

### 

#### Crystal data

[Cu(C_2_H_2_ClO_2_)_2_(C_8_H_7_NO)_2_(CH_4_O)]
*M* *_r_* = 548.86
Monoclinic, 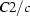

*a* = 19.860 (3) Å
*b* = 15.576 (3) Å
*c* = 15.017 (3) Å
β = 97.917 (3)°
*V* = 4600.9 (15) Å^3^
*Z* = 8
Mo *K*α radiation
μ = 1.23 mm^−1^
*T* = 173 (2) K
0.26 × 0.20 × 0.16 mm

#### Data collection

Nonius KappaCCD area-detector diffractometer
Absorption correction: multi-scan (*SORTAV*; Blessing, 1995 ▶) *T*_min_ = 0.776, *T*_max_ = 0.891 (expected range = 0.716–0.822)
16690 measured reflections
4021 independent reflections
3093 reflections with *I* > 2σ(*I*)
*R*_int_ = 0.076

#### Refinement

*R*[*F* ^2^ > 2σ(*F* ^2^)] = 0.043
*wR*(*F* ^2^) = 0.103
*S* = 1.02
4021 reflections
300 parameters
H-atom parameters constrained
Δρ_max_ = 1.08 e Å^−3^
Δρ_min_ = −0.53 e Å^−3^

### 

Data collection: *COLLECT* (Nonius, 1998 ▶); cell refinement: *SCALEPACK* (Otwinowski & Minor, 1997 ▶); data reduction: *DENZO* (Otwinowski & Minor, 1997 ▶) and *SCALEPACK*; program(s) used to solve structure: *SHELXS97* (Sheldrick, 2008 ▶); program(s) used to refine structure: *SHELXL97* (Sheldrick, 2008 ▶); molecular graphics: *ORTEP-3 for Windows* (Farrugia, 1997 ▶); software used to prepare material for publication: *enCIFer* (Allen *et al.*, 2004 ▶).

## Supplementary Material

Crystal structure: contains datablocks global, I. DOI: 10.1107/S1600536808008404/om2220sup1.cif

Structure factors: contains datablocks I. DOI: 10.1107/S1600536808008404/om2220Isup2.hkl

Additional supplementary materials:  crystallographic information; 3D view; checkCIF report

## Figures and Tables

**Table 1:** Selected bond lengths (Å)

Cu1—O8	1.956 (2)
Cu1—O4	1.964 (2)
Cu1—N21	2.031 (3)
Cu1—N11	2.046 (3)
Cu1—O1	2.311 (2)
Cu1—O5	2.833 (2)

**Table 2:** Hydrogen-bond geometry (Å, °)

*D*—H⋯*A*	*D*—H	H⋯*A*	*D*⋯*A*	*D*—H⋯*A*
O1—H1*O*⋯O9	0.84	1.86	2.664 (3)	159
C12—H12⋯O4	0.95	2.44	2.944 (4)	113
C20—H20⋯O8	0.95	2.40	2.915 (4)	114
C22—H22⋯O8	0.95	2.41	2.886 (4)	111
C30—H30⋯O4	0.95	2.53	3.000 (4)	111
C6—H6*A*⋯O5^i^	0.99	2.60	3.510 (4)	154
C16—H16*C*⋯O1^ii^	0.98	2.63	3.413 (4)	137
C14—H14⋯O9^ii^	0.95	2.55	3.450 (4)	159
C20—H20⋯O5^i^	0.95	2.57	3.221 (4)	126
C29—H29⋯O5^iii^	0.95	2.69	3.451 (4)	138
C19—H19⋯O5^i^	0.95	2.65	3.235 (4)	120
C16—H16*A*⋯O9^iv^	0.98	2.65	3.610 (4)	167
C24—H24⋯O9^v^	0.95	2.67	3.408 (4)	135
C1—H1*B*⋯C14^vi^	0.98	2.87	3.834 (4)	168

Symmetry codes: (i) 
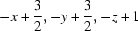
; (ii) 
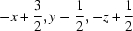
; (iii) 
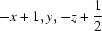
; (iv) 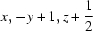; (v) 
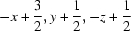
; (vi) 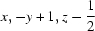.

**Fig. 1.**
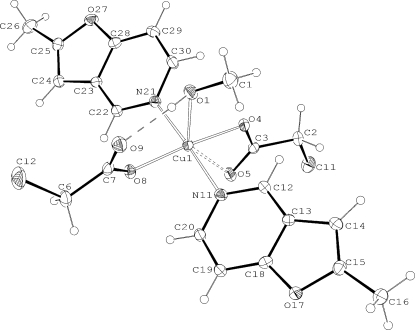
Perspective view of the title compound, with the atom numbering scheme and thermal ellipsoids drawn at the 30% probability level.

**Fig. 2.**
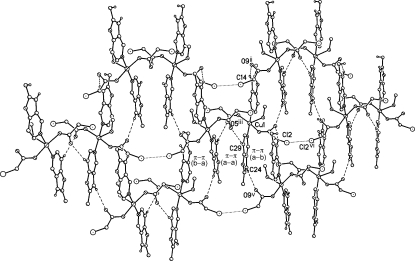
The crystal packing of the title compound with C–H···O hydrogen bonds, Cl···Cl contacts and π-π stacking interactions. Symmetry codes: (ii) -x + 3/2, y - 1/2, -z + 1/2; (iii) -x + 1, y, -z + 1/2; (v) -x + 3/2, y + 1/2, -z + 1/2; (vi) -x + 2, y, -z + 1/2].

**Fig. 3.**
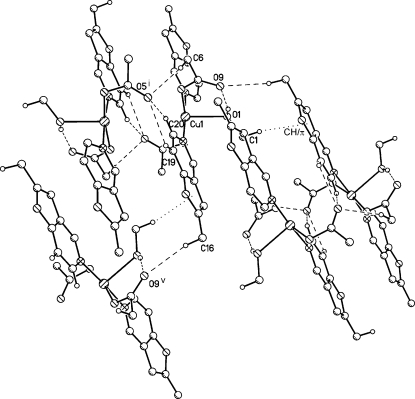
The C–H···O hydrogen bonds and CH/π interactions in crystal structure of the title compound. Symmetry codes: (i) -x + 3/2, -y + 3/2, -z + 1; (v) -x + 3/2, y + 1/2, -z + 1/2].

## supplementary crystallographic information

### Comment

Furopyridines are components of many biologically active compounds, for example the furo[3,2-*c*]pyridine ring system has potential antipsychotic activity (New *et al.*, 1989). The furo[3,2-*c*]pyridine and its derivatives can be readily coordinated to metal centers through the pyridine N-donor atom (Baran *et al.*, 2005; Ivaniková *et al.*, 2006; Mikloš *et al.* (2005); Miklovič *et al.*, 2004; Segľa *et al.* (2005); Titiš *et al.*, 2007; Vrábel *et al.*, 2007*a*,b). As part of our efforts to investigate metal(II) complexes based on furo[3,2-*c*]pyridine derivatives, we describe the X-ray characterization of the title compound.

In the title compound, the Cu^II^ atom is six-coordinated by two carboxylate O atoms of the asymmetrically chelating bidentate chloroacetate anion [Cu1–O4 = 1.964 (2) and Cu–O5 = 2.833 (2) Å], one carboxylate O atom of the monodentate chloroacetate anion [Cu1–O8 = 1.956 (2) Å], two N atoms of pyridine rings of 2-methylfuro[3,2-*c*]pyridine [Cu1–N11 = 2.046 (3) and Cu1–N21 = 2.031 (3) Å] and one O atom of methanol molecule [Cu1–O1 = 2.311 (2) Å], resulting in highly distorted square-bipyramidal geometry (Fig. 1). The intramolecular O–H···O hydrogen bond forms a six-membered metallocycle (Fig. 1).

The bond lengths and angles may be compared with the corresponding values in similar complexes, with axial water molecule [aquabis(benzoato)bis(γ-picoline)copper(II) (II) (refcode: BZGPCU10, Borel *et al.*, 1978); aquabis(3-pyridylacrylato)bis(3-pyridylmethanol)copper(II) (III), (refcode: XEYTAX, Moncol *et al.*, 2007); aquabis(acetato)bis(2-(3-pyridyl)-5-(4-pyridyl)-1,3,4-oxadiazole)copper(II) (IV), (refcode: QAQNOM, Wang *et al.*, 2005)]. In the molecular structure of all three complexes (II–IV), there is highly distorted square-bipyramidal (4 + 1 + 1) coordination environment, the longer Cu–O bond distances for asymmetrically chelating bidentate carboxylate anions are in the range of 2.61–2.78 Å.

The hydrogen-bond parameters of the title compound are listed in Table 2. The molecules of the title compound are linked through weak C–H···O hydrogen-bonding interactions (Figures 2 and 3), where acceptor atoms of hydrogen-bonds are carboxylate O atoms (O5 and O9). As can be seen in Figure 2, there are observed also short Cl2···Cl2^vi^ [symmetry code: (vi) -*x* + 2, *y*, -*z* + 1/2] contacts (Desiraju, 1995) of 3.384 (2)Å between the molecules of the title compound. The additional interactions are the π-π stacking interactions (Janiak, 2000), between the two adjacent furo[3,2-*c*]pyridine rings, [N21/C22—C25/O27/C28—C30] (π_a_) and [N11/C12—C15/O17/C18—C20] (π_b_). Four 2-methylfuro[3,2-*c*]pyridine rings are stacked in the order π_b_-π_a_···π_a_-π_a_···π_a_-π_b_ (Figure 2). The distances between π_a_-π_a_^iii^ and π_a_-π_b_^vii^ [symmetry codes: (iii) -*x* + 1, *y*, -*z* + 1/2, (vii) -*x* + 3/2, *y* + 1/2, -*z* + 1/2] 2-methylfuro[3,2-*c*]pyridine rings are in ranges 3.44–3.66 Å and 3.45–3.83 Å, respectively. The CH/π interaction (Suezawa *et al.*, 2002) is also observed between methyl H atom of the methanol ligand and furan ring of 2-methylfuro[3,2-*c*]pyridine [C1–H1B···π_b_^viii^, symmetry code: (viii) *x*, -*y* + 1, *z* - 1/2] (Figure 3). The distances *D*_atm_ (interatomic distance H1B/C14) and *D*_pln_ (H/π-plane distance) (Suezawa *et al.*, 2002) are 2.77 and 2.85 Å, respectively.

### Experimental

The organic compounds 2-methylfuro[3,2-*c*]pyridine (Mefpy) has been prepared using procedure described in Eloy & Deryckere (1971). Complex Cu(C_2_H_2_ClO_2_)_2_.2H_2_O (0.002 mol, 0.57 g) was dissolved in 30 cm^3^ of methanol and treated with a methanolic solution of Mefpy (0.004 mol, 0.53 g, 10 cm^3^ me thanol) in a molar ratio of 1:2. The mixture was stirred and left to stand at room temperature giving a crystalline compound of [Cu(C_2_H_2_ClO_2_)_2_(C_8_H_7_NO)_2_(CH_4_O)]. The Anal. Calc.: C, 45.95; H, 4.04; N, 5.10; Cu, 11.58; Found: C, 45.57; H, 3.87; N, 5.00; Cu, 11.45. IR (KBr) cm^-1^: 1640vs,br ν_as_(COO^-^); 1371vs ν_s_(COO^-^); 1605*m*ν(C?N)_Mefpy_; 654*m*δ(py)_Mefpy_; 428*m*χ(py)_Mefpy_; 1041*m*ν(*C*–*O*)_methanol_. UV-VIS: 645 nm.

### Refinement

All H atoms of C–H (aromatic, methyl and methylene) and hydroxyl O–H bonds were placed in calculated positions (0.95, 0.98, 0.99 and 0.84 Å, respectively); isotropic displacement parameters were fixed (*U*_iso_(H) = *xU*_iso_(C/O) (*x* = 1.2 for aromatic and methylene; and 1.5 for methyl and hydroxyl) of C or O atoms to which they were attached) using a riding model.

### Crystal data

**Table:**

[Cu(C_2_H_2_ClO_2_)_2_(C_8_H_7_NO)_2_(CH_4_O)]	*F*_000_ = 2248
*M**_r_* = 548.86	*D*_x_ = 1.585 Mg m^−^^3^
Monoclinic, *C*2/*c*	Mo *K*α radiation λ = 0.71073 Å
Hall symbol: -C2yc	Cell parameters from 4021 reflections
*a* = 19.860 (3) Å	θ = 2.3–25.0º
*b* = 15.576 (3) Å	µ = 1.23 mm^−^^1^
*c* = 15.017 (3) Å	*T* = 173 (2) K
β = 97.917 (3)º	Block, green
*V* = 4600.9 (15) Å^3^	0.26 × 0.20 × 0.16 mm
*Z* = 8	

### Data collection

**Table:**

Nonius KappaCCD area-detector diffractometer	4021 independent reflections
Radiation source: Enraf–Nonius FR590	3093 reflections with *I* > 2σ(*I*)
Monochromator: graphite	*R*_int_ = 0.076
Detector resolution: 9 pixels mm^-1^	θ_max_ = 25.0º
*T* = 173(2) K	θ_min_ = 2.3º
ω and φ scans	*h* = −21→23
Absorption correction: multi-scan(SORTAV; Blessing, 1995)	*k* = −16→18
*T*_min_ = 0.776, *T*_max_ = 0.891	*l* = −16→17
16690 measured reflections	

### Refinement

**Table:**

Refinement on *F*^2^	Secondary atom site location: difference Fourier map
Least-squares matrix: full	Hydrogen site location: inferred from neighbouring sites
*R*[*F*^2^ > 2σ(*F*^2^)] = 0.043	H-atom parameters constrained
*wR*(*F*^2^) = 0.103	*w* = 1/[σ^2^(*F*_o_^2^) + (0.0452*P*)^2^ + 8.8488*P*] where *P* = (*F*_o_^2^ + 2*F*_c_^2^)/3
*S* = 1.02	(Δ/σ)_max_ = 0.001
4021 reflections	Δρ_max_ = 1.08 e Å^−^^3^
300 parameters	Δρ_min_ = −0.53 e Å^−^^3^
Primary atom site location: structure-invariant direct methods	Extinction correction: none

### Special details

**Table:**

Geometry. All e.s.d.'s (except the e.s.d. in the dihedral angle between two l.s. planes) are estimated using the full covariance matrix. The cell e.s.d.'s are taken into account individually in the estimation of e.s.d.'s in distances, angles and torsion angles; correlations between e.s.d.'s in cell parameters are only used when they are defined by crystal symmetry. An approximate (isotropic) treatment of cell e.s.d.'s is used for estimating e.s.d.'s involving l.s. planes.
Refinement. Refinement of *F*^2^ against ALL reflections. The weighted *R*-factor *wR* and goodness of fit *S* are based on *F*^2^, conventional *R*-factors *R* are based on *F*, with *F* set to zero for negative *F*^2^. The threshold expression of *F*^2^ > σ(*F*^2^) is used only for calculating *R*-factors(gt) *etc*. and is not relevant to the choice of reflections for refinement. *R*-factors based on *F*^2^ are statistically about twice as large as those based on *F*, and *R*- factors based on ALL data will be even larger.

### Fractional atomic coordinates and isotropic or equivalent isotropic displacement parameters (Å^2^)

**Table:**

	*x*	*y*	*z*	*U*_iso_*/*U*_eq_	
Cu1	0.68144 (2)	0.73110 (3)	0.32031 (2)	0.02193 (13)	
Cl1	0.47011 (5)	0.61664 (6)	0.48363 (6)	0.0391 (2)	
Cl2	0.91758 (6)	0.91134 (8)	0.26724 (8)	0.0570 (3)	
O1	0.70672 (12)	0.67987 (16)	0.18435 (15)	0.0311 (6)	
H1O	0.7475	0.6953	0.1961	0.042*	
O4	0.60027 (12)	0.65827 (15)	0.31521 (14)	0.0245 (5)	
O5	0.58725 (13)	0.72145 (16)	0.44549 (15)	0.0329 (6)	
O8	0.75801 (11)	0.81089 (15)	0.34186 (15)	0.0255 (5)	
O9	0.82188 (13)	0.76344 (17)	0.24003 (16)	0.0346 (6)	
O17	0.85280 (12)	0.44366 (15)	0.51707 (14)	0.0260 (5)	
O27	0.51950 (12)	1.02340 (16)	0.12018 (15)	0.0308 (6)	
N11	0.73795 (14)	0.63899 (18)	0.39349 (17)	0.0239 (6)	
N21	0.62599 (14)	0.82756 (18)	0.25534 (17)	0.0234 (6)	
C1	0.7054 (2)	0.5932 (3)	0.1550 (3)	0.0401 (10)	
H1A	0.7410	0.5605	0.1921	0.060*	
H1B	0.7131	0.5908	0.0920	0.060*	
H1C	0.6609	0.5684	0.1609	0.060*	
C2	0.51644 (18)	0.6003 (2)	0.3912 (2)	0.0281 (8)	
H2A	0.5376	0.5426	0.3965	0.034*	
H2B	0.4844	0.6017	0.3346	0.034*	
C3	0.57154 (17)	0.6670 (2)	0.3861 (2)	0.0241 (7)	
C6	0.86409 (19)	0.8740 (2)	0.3437 (2)	0.0336 (9)	
H6A	0.8924	0.8462	0.3951	0.040*	
H6B	0.8411	0.9237	0.3674	0.040*	
C7	0.81103 (17)	0.8109 (2)	0.3028 (2)	0.0261 (8)	
C12	0.72171 (17)	0.5554 (2)	0.3792 (2)	0.0249 (7)	
H12	0.6819	0.5407	0.3395	0.030*	
C13	0.76207 (17)	0.4910 (2)	0.4212 (2)	0.0232 (7)	
C14	0.76156 (18)	0.3984 (2)	0.4209 (2)	0.0275 (8)	
H14	0.7293	0.3623	0.3866	0.033*	
C15	0.81551 (18)	0.3731 (2)	0.4786 (2)	0.0266 (8)	
C16	0.84283 (19)	0.2884 (2)	0.5111 (2)	0.0315 (8)	
H16A	0.8411	0.2836	0.5759	0.047*	
H16B	0.8900	0.2830	0.4996	0.047*	
H16C	0.8154	0.2426	0.4794	0.047*	
C18	0.81968 (17)	0.5148 (2)	0.4800 (2)	0.0244 (7)	
C19	0.83756 (17)	0.5989 (2)	0.4963 (2)	0.0243 (7)	
H19	0.8771	0.6147	0.5360	0.029*	
C20	0.79452 (17)	0.6590 (2)	0.4514 (2)	0.0238 (7)	
H20	0.8051	0.7180	0.4617	0.029*	
C22	0.63954 (17)	0.9106 (2)	0.2739 (2)	0.0246 (7)	
H22	0.6757	0.9250	0.3197	0.030*	
C23	0.60218 (17)	0.9759 (2)	0.2279 (2)	0.0226 (7)	
C24	0.60092 (18)	1.0681 (2)	0.2304 (2)	0.0263 (8)	
H24	0.6297	1.1042	0.2701	0.032*	
C25	0.55152 (18)	1.0935 (2)	0.1662 (2)	0.0275 (8)	
C26	0.5241 (2)	1.1790 (3)	0.1366 (3)	0.0374 (9)	
H26A	0.5440	1.2229	0.1791	0.056*	
H26B	0.4746	1.1790	0.1347	0.056*	
H26C	0.5356	1.1917	0.0766	0.056*	
C28	0.55083 (18)	0.9522 (2)	0.1590 (2)	0.0266 (8)	
C29	0.53615 (18)	0.8676 (2)	0.1379 (2)	0.0301 (8)	
H29	0.5011	0.8515	0.0913	0.036*	
C30	0.57544 (18)	0.8075 (2)	0.1887 (2)	0.0282 (8)	
H30	0.5665	0.7485	0.1761	0.034*	

### Atomic displacement parameters (Å^2^)

**Table:**

	*U*^11^	*U*^22^	*U*^33^	*U*^12^	*U*^13^	*U*^23^
Cu1	0.0215 (2)	0.0189 (2)	0.0251 (2)	−0.00278 (18)	0.00206 (15)	0.00040 (16)
Cl1	0.0352 (5)	0.0397 (6)	0.0453 (6)	−0.0002 (4)	0.0153 (4)	0.0061 (4)
Cl2	0.0403 (6)	0.0577 (8)	0.0748 (8)	−0.0195 (6)	0.0142 (5)	0.0131 (6)
O1	0.0346 (15)	0.0283 (15)	0.0303 (13)	−0.0045 (12)	0.0037 (10)	−0.0056 (10)
O4	0.0244 (13)	0.0213 (13)	0.0276 (13)	−0.0045 (10)	0.0031 (9)	−0.0003 (9)
O5	0.0376 (15)	0.0274 (15)	0.0333 (14)	−0.0084 (12)	0.0033 (11)	−0.0046 (11)
O8	0.0220 (13)	0.0193 (13)	0.0346 (13)	−0.0003 (10)	0.0023 (10)	0.0006 (10)
O9	0.0335 (15)	0.0354 (15)	0.0353 (14)	−0.0043 (12)	0.0065 (11)	−0.0019 (11)
O17	0.0273 (13)	0.0228 (13)	0.0265 (12)	−0.0005 (11)	−0.0017 (9)	0.0015 (10)
O27	0.0319 (14)	0.0308 (15)	0.0277 (13)	0.0050 (12)	−0.0028 (10)	0.0045 (10)
N11	0.0246 (16)	0.0272 (17)	0.0199 (14)	−0.0026 (13)	0.0030 (11)	−0.0017 (11)
N21	0.0215 (15)	0.0263 (17)	0.0224 (14)	−0.0026 (12)	0.0036 (11)	0.0001 (11)
C1	0.051 (3)	0.030 (2)	0.040 (2)	−0.0026 (19)	0.0080 (18)	−0.0042 (17)
C2	0.028 (2)	0.0220 (19)	0.0347 (19)	−0.0048 (16)	0.0075 (15)	−0.0013 (14)
C3	0.0235 (19)	0.0196 (19)	0.0277 (19)	0.0012 (14)	−0.0017 (14)	0.0036 (14)
C6	0.026 (2)	0.029 (2)	0.046 (2)	−0.0072 (16)	0.0048 (16)	0.0026 (16)
C7	0.0230 (19)	0.0218 (19)	0.032 (2)	0.0004 (15)	−0.0009 (15)	0.0089 (15)
C12	0.0256 (19)	0.024 (2)	0.0245 (17)	−0.0046 (15)	0.0002 (13)	−0.0020 (14)
C13	0.0274 (19)	0.0249 (19)	0.0171 (16)	−0.0026 (15)	0.0020 (13)	−0.0008 (13)
C14	0.031 (2)	0.026 (2)	0.0242 (18)	−0.0025 (16)	−0.0014 (14)	−0.0038 (14)
C15	0.035 (2)	0.0222 (19)	0.0237 (18)	0.0001 (16)	0.0072 (14)	−0.0005 (13)
C16	0.034 (2)	0.027 (2)	0.034 (2)	0.0018 (16)	0.0025 (15)	0.0015 (15)
C18	0.0265 (19)	0.027 (2)	0.0197 (17)	0.0022 (16)	0.0033 (13)	0.0028 (13)
C19	0.0238 (19)	0.025 (2)	0.0236 (17)	−0.0045 (15)	0.0007 (13)	−0.0014 (13)
C20	0.0247 (19)	0.0228 (19)	0.0239 (17)	−0.0055 (15)	0.0030 (13)	−0.0022 (13)
C22	0.0252 (19)	0.025 (2)	0.0235 (17)	−0.0052 (15)	0.0030 (13)	−0.0008 (13)
C23	0.0227 (18)	0.0249 (19)	0.0196 (17)	−0.0006 (15)	0.0012 (12)	0.0015 (13)
C24	0.0263 (19)	0.024 (2)	0.0282 (18)	−0.0022 (15)	0.0024 (14)	0.0000 (14)
C25	0.026 (2)	0.027 (2)	0.0302 (19)	−0.0004 (16)	0.0081 (15)	0.0017 (14)
C26	0.038 (2)	0.035 (2)	0.038 (2)	0.0091 (19)	0.0036 (17)	0.0105 (16)
C28	0.0270 (19)	0.031 (2)	0.0220 (18)	0.0021 (16)	0.0052 (14)	0.0024 (14)
C29	0.026 (2)	0.034 (2)	0.0280 (19)	−0.0029 (16)	−0.0042 (14)	−0.0020 (15)
C30	0.028 (2)	0.027 (2)	0.0298 (19)	−0.0072 (16)	0.0023 (14)	−0.0049 (15)

### Geometric parameters (Å, °)

**Table:**

Cu1—O8	1.956 (2)	C14—C15	1.341 (5)
Cu1—O4	1.964 (2)	C14—H14	0.9500
Cu1—N21	2.031 (3)	C15—O17	1.404 (4)
Cu1—N11	2.046 (3)	C15—C16	1.484 (5)
Cu1—O1	2.311 (2)	C16—H16A	0.9800
Cu1—O5	2.833 (2)	C16—H16B	0.9800
Cl1—C2	1.786 (3)	C16—H16C	0.9800
Cl2—C6	1.767 (4)	O17—C18	1.367 (4)
Cl2—Cl2^i^	3.384 (2)	C18—C19	1.371 (5)
O1—C1	1.419 (4)	C19—C20	1.380 (5)
O1—H1O	0.84	C19—H19	0.9500
C1—H1A	0.9800	C20—H20	0.9500
C1—H1B	0.9800	N21—C22	1.343 (4)
C1—H1C	0.9800	N21—C30	1.353 (4)
O4—C3	1.282 (4)	C22—C23	1.385 (5)
O5—C3	1.239 (4)	C22—H22	0.9500
C2—C3	1.519 (5)	C23—C28	1.399 (5)
C2—H2A	0.9900	C23—C24	1.438 (5)
C2—H2B	0.9900	C24—C25	1.337 (5)
O8—C7	1.273 (4)	C24—H24	0.9500
O9—C7	1.240 (4)	C25—O27	1.398 (4)
C6—C7	1.509 (5)	C25—C26	1.483 (5)
C6—H6A	0.9900	C26—H26A	0.9800
C6—H6B	0.9900	C26—H26B	0.9800
N11—C12	1.351 (4)	C26—H26C	0.9800
N11—C20	1.359 (4)	O27—C28	1.362 (4)
C12—C13	1.382 (5)	C28—C29	1.378 (5)
C12—H12	0.9500	C29—C30	1.379 (5)
C13—C18	1.396 (5)	C29—H29	0.9500
C13—C14	1.442 (5)	C30—H30	0.9500
			
O8—Cu1—O4	171.36 (9)	C15—C14—C13	106.7 (3)
O8—Cu1—N21	88.12 (10)	C15—C14—H14	126.7
O4—Cu1—N21	91.19 (10)	C13—C14—H14	126.7
O8—Cu1—N11	90.01 (10)	C14—C15—O17	111.4 (3)
O4—Cu1—N11	90.14 (10)	C14—C15—C16	134.2 (3)
N21—Cu1—N11	176.10 (10)	O17—C15—C16	114.3 (3)
O8—Cu1—O1	96.14 (9)	C15—C16—H16A	109.5
O4—Cu1—O1	92.47 (9)	C15—C16—H16B	109.5
N21—Cu1—O1	90.02 (10)	H16A—C16—H16B	109.5
N11—Cu1—O1	93.59 (10)	C15—C16—H16C	109.5
O8—Cu1—O5	119.56 (8)	H16A—C16—H16C	109.5
O4—Cu1—O5	51.81 (8)	H16B—C16—H16C	109.5
N21—Cu1—O5	89.66 (9)	C18—O17—C15	105.6 (3)
N11—Cu1—O5	88.28 (9)	O17—C18—C19	127.1 (3)
O1—Cu1—O5	144.26 (8)	O17—C18—C13	110.4 (3)
C1—O1—Cu1	127.3 (2)	C19—C18—C13	122.4 (3)
C1—O1—H1O	108.3	C18—C19—C20	115.7 (3)
Cu1—O1—H1O	92.0	C18—C19—H19	122.1
O1—C1—H1A	109.8	C20—C19—H19	122.1
O1—C1—H1B	109.7	N11—C20—C19	124.0 (3)
H1A—C1—H1B	109.5	N11—C20—H20	118.0
O1—C1—H1C	109.0	C19—C20—H20	118.0
H1A—C1—H1C	109.5	C22—N21—C30	118.9 (3)
H1B—C1—H1C	109.5	C22—N21—Cu1	122.2 (2)
C3—O4—Cu1	111.3 (2)	C30—N21—Cu1	118.8 (2)
C3—O5—Cu1	71.48 (19)	N21—C22—C23	121.7 (3)
C3—C2—Cl1	113.2 (2)	N21—C22—H22	119.1
C3—C2—H2A	108.9	C23—C22—H22	119.1
Cl1—C2—H2A	108.9	C22—C23—C28	117.5 (3)
C3—C2—H2B	108.9	C22—C23—C24	136.9 (3)
Cl1—C2—H2B	108.9	C28—C23—C24	105.6 (3)
H2A—C2—H2B	107.8	C25—C24—C23	106.9 (3)
O5—C3—O4	125.0 (3)	C25—C24—H24	126.6
O5—C3—C2	122.9 (3)	C23—C24—H24	126.6
O4—C3—C2	112.1 (3)	C24—C25—O27	111.4 (3)
C7—O8—Cu1	126.7 (2)	C24—C25—C26	133.1 (3)
C7—C6—Cl2	113.5 (3)	O27—C25—C26	115.5 (3)
C7—C6—H6A	108.8	C25—C26—H26A	109.5
Cl2—C6—H6A	108.8	C25—C26—H26B	109.5
C7—C6—H6B	109.0	H26A—C26—H26B	109.5
Cl2—C6—H6B	108.9	C25—C26—H26C	109.5
H6A—C6—H6B	107.7	H26A—C26—H26C	109.5
O9—C7—O8	126.2 (3)	H26B—C26—H26C	109.5
O9—C7—C6	120.9 (3)	C28—O27—C25	105.9 (3)
O8—C7—C6	112.8 (3)	O27—C28—C29	127.7 (3)
C12—N11—C20	118.8 (3)	O27—C28—C23	110.2 (3)
C12—N11—Cu1	119.3 (2)	C29—C28—C23	122.1 (3)
C20—N11—Cu1	121.8 (2)	C28—C29—C30	115.9 (3)
N11—C12—C13	121.1 (3)	C28—C29—H29	122.0
N11—C12—H12	119.5	C30—C29—H29	122.0
C13—C12—H12	119.5	N21—C30—C29	123.9 (3)
C12—C13—C18	118.0 (3)	N21—C30—H30	118.1
C12—C13—C14	136.2 (3)	C29—C30—H30	118.1
C18—C13—C14	105.8 (3)		
			
O8—Cu1—O1—C1	136.2 (3)	C13—C14—C15—C16	177.9 (4)
O4—Cu1—O1—C1	−44.5 (3)	C14—C15—O17—C18	−0.4 (4)
N21—Cu1—O1—C1	−135.7 (3)	C16—C15—O17—C18	−179.2 (3)
N11—Cu1—O1—C1	45.8 (3)	C15—O17—C18—C19	−179.3 (3)
O5—Cu1—O1—C1	−46.2 (3)	C15—O17—C18—C13	1.3 (3)
N21—Cu1—O4—C3	−92.4 (2)	C12—C13—C18—O17	178.7 (3)
N11—Cu1—O4—C3	83.9 (2)	C14—C13—C18—O17	−1.7 (4)
O1—Cu1—O4—C3	177.5 (2)	C12—C13—C18—C19	−0.7 (5)
O5—Cu1—O4—C3	−3.75 (19)	C14—C13—C18—C19	178.9 (3)
O8—Cu1—O5—C3	−176.77 (19)	O17—C18—C19—C20	−178.7 (3)
O4—Cu1—O5—C3	3.81 (19)	C13—C18—C19—C20	0.6 (5)
N21—Cu1—O5—C3	95.6 (2)	C12—N11—C20—C19	0.9 (5)
N11—Cu1—O5—C3	−87.7 (2)	Cu1—N11—C20—C19	−174.1 (2)
O1—Cu1—O5—C3	6.0 (3)	C18—C19—C20—N11	−0.7 (5)
Cu1—O5—C3—O4	−5.6 (3)	O8—Cu1—N21—C22	−26.3 (3)
Cu1—O5—C3—C2	172.3 (3)	O4—Cu1—N21—C22	145.1 (3)
Cu1—O4—C3—O5	8.2 (4)	O1—Cu1—N21—C22	−122.5 (3)
Cu1—O4—C3—C2	−169.9 (2)	O5—Cu1—N21—C22	93.3 (3)
Cl1—C2—C3—O5	7.2 (4)	O8—Cu1—N21—C30	151.0 (2)
Cl1—C2—C3—O4	−174.6 (2)	O4—Cu1—N21—C30	−37.6 (2)
N21—Cu1—O8—C7	−106.4 (3)	O1—Cu1—N21—C30	54.8 (2)
N11—Cu1—O8—C7	77.0 (3)	O5—Cu1—N21—C30	−89.4 (2)
O1—Cu1—O8—C7	−16.6 (3)	C30—N21—C22—C23	1.5 (5)
O5—Cu1—O8—C7	165.0 (2)	Cu1—N21—C22—C23	178.7 (2)
Cl2^i^—Cl2—C6—C7	−117.3 (3)	N21—C22—C23—C28	−2.0 (5)
Cu1—O8—C7—O9	4.8 (5)	N21—C22—C23—C24	178.3 (4)
Cu1—O8—C7—C6	−172.6 (2)	C22—C23—C24—C25	179.6 (4)
Cl2—C6—C7—O9	29.4 (4)	C28—C23—C24—C25	0.0 (4)
Cl2—C6—C7—O8	−153.1 (3)	C23—C24—C25—O27	−0.2 (4)
O8—Cu1—N11—C12	−156.7 (2)	C23—C24—C25—C26	178.3 (4)
O4—Cu1—N11—C12	31.9 (2)	C24—C25—O27—C28	0.4 (4)
O1—Cu1—N11—C12	−60.6 (2)	C26—C25—O27—C28	−178.4 (3)
O5—Cu1—N11—C12	83.7 (2)	C25—O27—C28—C29	178.6 (3)
O8—Cu1—N11—C20	18.3 (3)	C25—O27—C28—C23	−0.4 (3)
O4—Cu1—N11—C20	−153.1 (2)	C22—C23—C28—O27	−179.4 (3)
O1—Cu1—N11—C20	114.4 (2)	C24—C23—C28—O27	0.3 (4)
O5—Cu1—N11—C20	−101.3 (2)	C22—C23—C28—C29	1.5 (5)
C20—N11—C12—C13	−1.0 (5)	C24—C23—C28—C29	−178.8 (3)
Cu1—N11—C12—C13	174.2 (2)	O27—C28—C29—C30	−179.3 (3)
N11—C12—C13—C18	0.9 (5)	C23—C28—C29—C30	−0.4 (5)
N11—C12—C13—C14	−178.6 (3)	C22—N21—C30—C29	−0.3 (5)
C12—C13—C14—C15	−179.1 (4)	Cu1—N21—C30—C29	−177.6 (3)
C18—C13—C14—C15	1.4 (4)	C28—C29—C30—N21	−0.3 (5)
C13—C14—C15—O17	−0.6 (4)		

Symmetry codes: (i) −*x*+2, *y*, −*z*+1/2.

### Hydrogen-bond geometry (Å, °)

**Table:**

*D*—H···*A*	*D*—H	H···*A*	*D*···*A*	*D*—H···*A*
O1—H1O···O9	0.84	1.86	2.664 (3)	159
C12—H12···O4	0.95	2.44	2.944 (4)	113
C20—H20···O8	0.95	2.40	2.915 (4)	114
C22—H22···O8	0.95	2.41	2.886 (4)	111
C30—H30···O4	0.95	2.53	3.000 (4)	111
C6—H6A···O5^ii^	0.99	2.60	3.510 (4)	154
C16—H16C···O1^iii^	0.98	2.63	3.413 (4)	137
C14—H14···O9^iii^	0.95	2.55	3.450 (4)	159
C20—H20···O5^ii^	0.95	2.57	3.221 (4)	126
C29—H29···O5^iv^	0.95	2.69	3.451 (4)	138
C19—H19···O5^ii^	0.95	2.65	3.235 (4)	120
C16—H16A···O9^v^	0.98	2.65	3.610 (4)	167
C24—H24···O9^vi^	0.95	2.67	3.408 (4)	135
C1—H1B···C14^vii^	0.98	2.87	3.834 (4)	168

Symmetry codes: (ii) −*x*+3/2, −*y*+3/2, −*z*+1; (iii) −*x*+3/2, *y*−1/2, −*z*+1/2; (iv) −*x*+1, *y*, −*z*+1/2; (v) *x*, −*y*+1, *z*+1/2; (vi) −*x*+3/2, *y*+1/2, −*z*+1/2; (vii) *x*, −*y*+1, *z*−1/2.
